# Development of a novel core genome MLST scheme for tracing multidrug resistant *Staphylococcus capitis*

**DOI:** 10.1038/s41467-022-31908-x

**Published:** 2022-07-22

**Authors:** Zhengan Wang, Chao Gu, Lu Sun, Feng Zhao, Ying Fu, Lingfang Di, Junxiong Zhang, Hemu Zhuang, Shengnan Jiang, Haiping Wang, Feiteng Zhu, Yiyi Chen, Mengzhen Chen, Xia Ling, Yan Chen, Yunsong Yu

**Affiliations:** 1 Department of Infectious Diseases, Sir Run Run Shaw Hospital, Zhejiang University School of Medicine, Hangzhou, China; 2 Key Laboratory of Microbial Technology and Bioinformatics of Zhejiang Province, Hangzhou, Zhejiang Province China; 3 Regional Medical Center for National Institute of Respiratory Diseases, Sir Run Run Shaw Hospital, Zhejiang University School of Medicine, Hangzhou, China; 4 Department of Respiratory Medicine, The First Hospital of Jiaxing (the Affiliated Hospital of Jiaxing University), 1882 South Zhonghuan Road, Jiaxing, Zhejiang Province China; 5 Department of Clinical Laboratory, Sir Run Run Shaw Hospital, Zhejiang University School of Medicine, Hangzhou, Zhejiang Province China; 6 Key Laboratory of Precision Medicine in Diagnosis and Monitoring Research of Zhejiang Province, Hangzhou, Zhejiang Province China; 7 Department of Clinical Laboratory, Tongxiang First people’s hospital, Tongxiang, Zhejiang Province China; 8 Department of Clinical Laboratory, Linping Traditional Chinese Medicine Hospital, Hangzhou, China; 9 Blood center of Zhejiang province, Hangzhou, Zhejiang Province China

**Keywords:** Genetics research, Bacterial genetics, Medical genomics, Bacterial infection, Antimicrobial resistance

## Abstract

*Staphylococcus capitis*, which causes bloodstream infections in neonatal intensive care units, is a common cause of healthcare-associated infections. Thus, a standardized high-resolution typing method to document the transmission and dissemination of multidrug-resistant *S. capitis* isolates is required. We aimed to establish a core genome multilocus sequence typing (cgMLST) scheme to surveil *S. capitis*. The cgMLST scheme was defined based on primary and validation genome sets and tested with outbreaks of linezolid-resistant isolates and a validation set. Phylogenetic analysis was performed to investigate the population structure and compare it with the result of cgMLST analysis. The *S. capitis* population consists of 1 dominant, NRCS-A, and 4 less common clones. In this work, a multidrug-resistant clone (L clone) with linezolid resistance is identified. With the features of type III SCC*mec* and multiple copies of mutations of G2576T and C2104T in the 23S rRNA, the L clone has been spreading silently across China.

## Abstract (web summary)

*Staphylococcus capitis* is a common causative agent of bloodstream infections in neonatal intensive care units, with multidrug resistant isolates complicating treatment. Authors aimed to establish a core genome multilocus sequence typing (cgMLST) scheme to document the transmission and dissemination of multidrug-resistant *S. capitis* isolates.

## Introduction

*Staphylococcus capitis*, a coagulase-negative *Staphylococcus* (CoNS), is one of the most widely distributed opportunistic pathogens. This organism causes a wide variety of diseases, including endocarditis, catheter-related bacteremia, prosthetic joint infections (PJIs), and skin infection^1,2^. In particular, it is known to cause nosocomial late-onset sepsis (LOS) in neonatal intensive care units (NICUs)^3,4^, which leads to increased rates of morbidity and mortality^5,6^. In some areas, *S. capitis* is the most frequently detected pathogen in NICUs infants, outranking even *S. epidermidis*^4^.

To date, only a few studies have investigated the population structure of *S. capitis*, except for the NRCS-A clone^7^, which has emerged as a major pathogen among newborns in NICUs and has been isolated in more than 17 countries throughout the world^8^. The NRCS-A clone was first reported by Rasigade et al. in 2012^4^, who collected 40 *S. capitis* isolates from several French NICUs. *Sma*I pulsed-field gel electrophoresis (PFGE) typing indicated that most isolates are clonally related and belong to the same clone, NRCS-A. Further studies have indicated that multidrug resistance, especially non-susceptibility to vancomycin, is an important advantage for epidemical success^7^ In addition to neonates in NICUs, this clone also causes healthcare-associated infections in adults such as PJIs^9^.

Phylogenetic analysis based on whole-genome sequencing and PFGE are the most frequently used methods to investigate the molecular epidemiology of *S. capitis*. However, PFGE is labor-intensive, whereas phylogenetic analysis requires expert phylogenetic knowledge and specialist hardware. The lack of reliable and easily applicable typing methods has resulted in an underestimation of the significance of *S. capitis* in the clinical spread of multidrug-resistant isolates.

With the development of whole-genome sequencing technology, the use of core genome multilocus sequence typing (cgMLST) to subtype and monitor outbreaks of bacteria is becoming more common. The typing ability of cgMLST has proven to be reliable for the typing of several pathogenic bacteria including *Staphylococcus aureus*^10^, *Staphylococcus epidermidis*^11^, *Streptococcus mutans*^12^, and *Klebsiella pneumoniae*^13^. The typing technology uses genome-wide gene-by-gene alleles from hundreds or thousands of genes conserved in all or most members of the species, and this confers the technology with a considerably higher resolution than that of PFGE^14,15^. Standardization is another important benefit of cgMLST. The standardized method, which can be easily performed using commercial software, makes it possible to compare the results among international laboratories. More importantly, cgMLST is a high-resolution, accessible, and replicable typing method to detect outbreaks and analyze the relationship between bacterial isolates. Stenmark et al. successfully applied cgMLST analysis in a surveillance project of clinical *S. capitis* isolates detecting the dissemination of NRCS-A clone from a Swedish NICU^16^, but this scheme is not publicly available and 1063 loci limited the discriminatory power.

The need for a standardized typing method is urgent, considering the emergence of multidrug-resistant clones, especially clones resistant to linezolid^17–19^. Linezolid-resistant *S. capitis* (LRSC) poses a serious threat to clinical practice. Linezolid resistance is associated with two major mechanisms: (1) mutation of the 23S rRNA or ribosomal proteins L3 and L4^20^; and (2) acquisition of resistance genes such as the chloramphenicol-florfenicol resistance (*cfr*) gene^21^. The expression of Cfr methyltransferase confers resistance to linezolid and other ribosome-targeting antibiotics, which is known as the PhLOPS_A_ resistance phenotype (resistance to oxazolidinones, phenicols, lincosamides, pleuromutilins, and streptogramin A)^22^.

Here, we aimed to establish a cgMLST scheme for *S. capitis*. We developed a process to document the transmission and dissemination of multidrug-resistant *S. capitis* isolates, which involved three major steps. First, we detected the initial core genes with the primary genome set. Second, we improved the core genome using a validation genome set. Third, we evaluated the cgMLST scheme using a test genome set. With the aid of the newly established cgMLST scheme, we identified a unique multidrug-resistant clone, the L clone, which is widely distributed in China.

## Results

### Establishment of the *S. capitis* cgMLST scheme

All available genome assemblies of *S. capitis* in public genome database were collected and served as primary genome set. The core genome analysis of the primary genome set identified 2077 genes as comprising the core genome. After applying seven exclusion criteria, 1826 genes were obtained as the basis for the primary cgMLST scheme. Subsequently, the validation set was typed with this scheme, and 334 genes having an error rate of greater than 5% were excluded. Most of the error reports were for alleles containing a frame shift. After discarding the erroneous genes from the primary cgMLST scheme, we obtained the final cgMLST scheme, consisting of 1492 genes with a total length of approximately 1.39 megabases. The genes in the final cgMLST scheme had an average length of 931.5 bp (standard deviation, 580.8 bp; range, 90–7170 bp), with mean ± standard deviation GC content equal to 33.6 ± 3.0%. Overall, 1491 genes were detected in the reference genome CR01 chromosome, excluding gene “group_1475”, covering 55.1% of the full genome. The core genes were evenly distributed across the genome (Supplementary Fig. 2).

### Evaluation and comparison of the *S. capitis* population structure using the cgMLST scheme

**Fig. 1:**
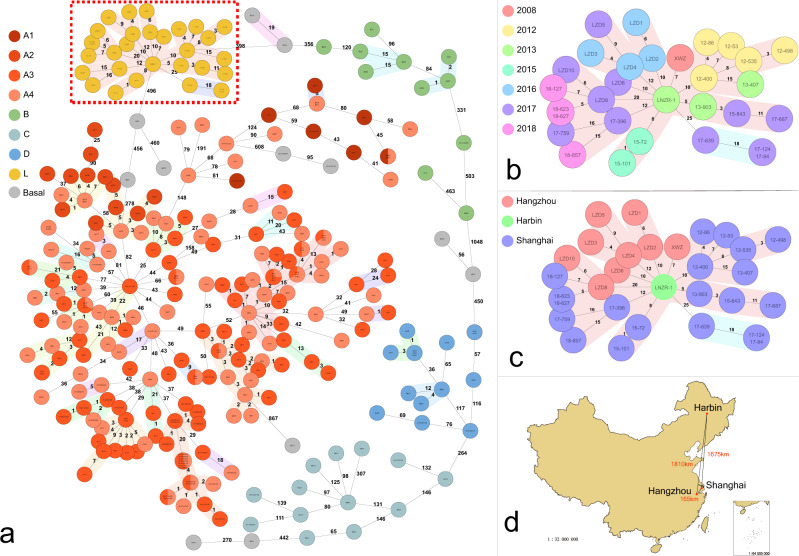
Minimum spanning tree of the validation set and the linezolid-resistant *Staphylococcus capitis* (LRSC) isolates. **a** Minimum spanning tree of the validation set and LRSC isolates using the core genome multilocus sequence typing (cgMLST) scheme. Groups were painted with different colors in the background. Nodes were painted with the same color according to the phylogenetic tree. **b**, **c** Enlarged image of the L clone, the region surrounded by a dotted frame in the minimum spanning tree. **b** is labeled with the year of isolation in different colors, whereas **c** is labeled with the city of isolation. **d** Map of China, showing the source of isolates labeled with a red node. The distances between cities are marked alongside the lines. Source data are provided as a Source Data file.

To evaluate the novel cgMLST scheme, the validation set containing 250 *S. capitis* genomes was used to create a minimum spanning tree with the default settings in Ridom (Ridom GmbH, Würzburg, Germany) (Fig. 1a). The cgMLST typing results showed that at least 95% of the target genes were present in all genomes (100%), with a median (interquartile range) of 99.87% (99.73–99.93%) of the 1492 target genes detected per genome. The number of non-typeable genes averaged to 3.2 ± 4.0 genes per genome (range, 0–24), which occurred mostly due to the absence of genes or early stop codons in those genes. The average number of alleles reported for each cgMLST target gene was 8.1 ± 3.7 (range, 1–30) alleles.

A total of 217 distinct cgMLST allelic profiles were identified for the 250 genomes (missing data disregarded in the pairwise comparisons), and only 21 profiles contained multiple genomes. Most genomes of the validation set (179 isolates) were separated into 35 related isolated groups. The largest four groups consisted of 29, 20, 15, and 10 genomes, respectively, and all belonged to clone NRCS-A.

**Fig. 2:**
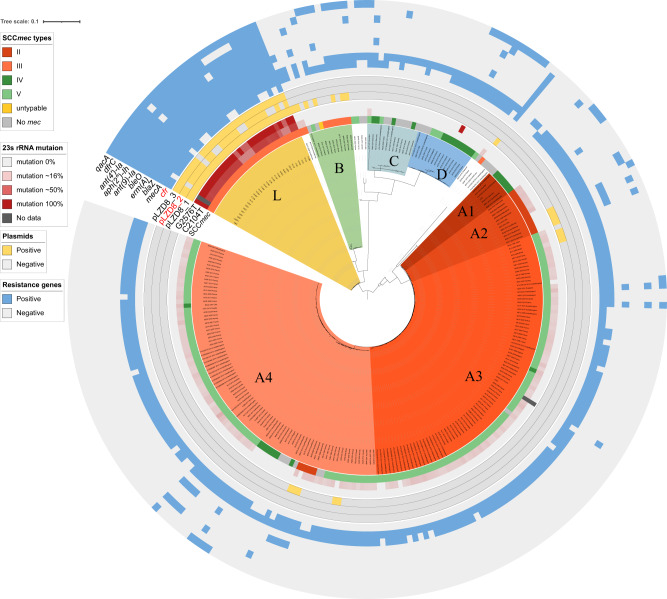
Phylogenetic tree of the validation set and the Linezolid-resistant *Staphylococcus capitis* (LRSC) isolates. The bootstraps are represented by the darkness of the line, and almost all of them were >95%. The clusters are labeled with uppercase letters in each clade and painted with different colors. The tree is surrounded with color strips, indicating the SCC*mec* type, percentage of G2576T and C2104T, distribution of plasmids, and distribution of antimicrobial resistance (AMR) genes, respectively. The shade of red in the 23s rRNA mutation represents the percentage of mutated copies, the lighter color indicates less mutation. The gene *cfr* and *cfr*-carrying plasmid are highlighted in red. The image with full information can be viewed in SVG format through this web link (https://itol.embl.de/export/122225230186443251631589900). Source data are provided as a Source Data file.

To compare the typing results of cgMLST with other sequence-based methods, an SNP-based phylogenetic analysis of the validation set was performed (Fig. 2). In total, 90,821 variable sites were identified in the alignment concatemer of the core genome. The number of distinct genotypes defined by SNPs was 242, which was nearly equivalent to the distinct profiles identified with cgMLST (*n* = 217), indicating that cgMLST and core genome SNP provided comparable resolution in the validation set.

The SNP phylogenetic tree provided greater discrimination than the minimum spanning tree. Based on the phylogenetic analysis, the population can be generally divided into four clusters based on monophyletic groups. The largest cluster A, representing the NRCS-A clone, was divided into four sub-clusters: A1, A2, A3, and A4. Compared to the result of a previous phylogenetic analysis^7^, subgroups A1 and A2 corresponded to proto-outbreaks 1 and 2, whereas A3 and A4 were subdivisions of cluster outbreak. Other isolates, known as basal clones, could be divided into three major groups, namely, clusters B, C, and D. Although clusters B, C, and D were clearly separated by the cgMLST analysis, the subgroups of the NRCS-A clone were mixed together in the minimum spanning tree.

### Application of cgMLST in LRSC isolates

We applied the cgMLST scheme to 31 LRSC isolates obtained from two independent outbreaks and sporadic cases. All 31 genomes were typeable and the median (interquartile range) of typeable genes was 99.93% (99.90–99.97%) (range, 99.3–100%). The 31 genomes were typed into 29 distinct cgMLST allele profiles, of which 30 genomes were closely related and formed two related groups. Excluding isolate LZD7, the largest allelic difference among the isolates was 25. The close genetic relationship among these LRSC isolates was also supported by the SNP-based phylogenetic analysis. These results were consistent with the results of the original publication of the outbreak analysis and indicated that a single *S. capitis* clone had spread around China. This was unexpected considering the tremendous geographical expanse of up to 1810 km and the wide time span, 10 years from 2008 to 2018 (Fig. 1b–d). Overall, we identified the clone having the feature of linezolid resistance and named it the L clone.

### Genetic and clinical characteristics of the L clone

Antimicrobial resistance gene were unevenly distributed among the clusters. In the validation set, 89.2% (223/250) of the isolates carried SCC*mec*, and clade A1 was type IV, A2 was type II, and most of A3 and A4 were type V. All isolates of L clone, carried SCC*mec* and considered as methicillin-resistant *S. capitis* (MRSC). Furthermore, the L clone gained more resistance genes than the other clusters, including genes *cfr*, *erm(A)*, *aph(2’)-Ih*, *ant(4’)-Ia*, *ant(9)-Ia*, *bleO*, and *dfrC*, which confer resistance to anti-ribosomal drugs, aminoglycoside, bleomycin, and sulfamethoxazole/trimethoprim (SMZ). Notably, all L clone isolates carried the *qacA* gene, which mediates resistance to quaternary ammonium compounds (Fig. 2). Except *cfr*, no other linezolid-resistance relevant genes, such as *optrA* and *poxtA*, were detected.

**Fig. 3:**
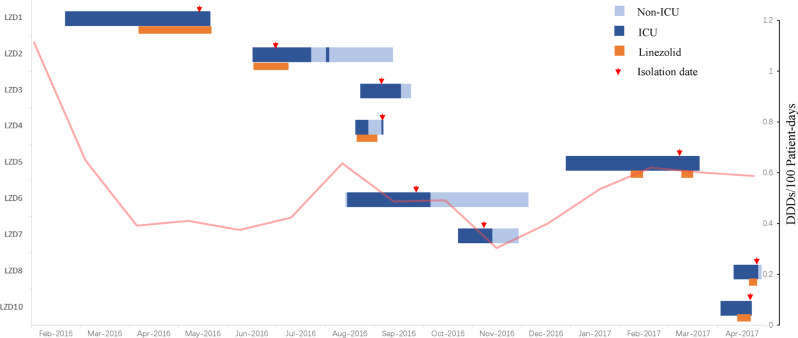
Timeline of the clinical cases. Nine clinical isolates were recovered from Sir Run Run Shaw Hospital. The blue rectangle represents the hospitalization progress, the dark blue rectangle indicates an intensive care unit (ICU) stay, and the light blue indicates a non-ICU stay. The red arrow indicates the isolation event. The light red line indicates the AUD of linezolid used in our hospital every month. Source data are provided as a Source Data file.

The outbreak of LRSC in Sir Run Run Shaw Hospital revealed the clinical characteristics of the L clone. All nine strains were isolated from patients with bacteremia (Table 1). All patients had associations with the intensive care unit (ICU) and had received antibiotics before the bacteremia episode, except for patients infected by LZD3, LZD6, and LZD7 (Fig. 3). During this period in our hospital, the AUD (antibiotics use density) every month ranged from 0.30 to 1.11 DDDs (defined daily dose per 100 patient-days) (Fig. 3). Susceptibility tests showed high levels of linezolid resistance (256 mg L^−1^), except in LZD6 and LZD7 (32 mg L^−1^) (Table 1). Besides resistance to linezolid, all isolates were methicillin resistant, with the cefoxitin MIC (minimum inhibitory concentration) of 128 mg L^−1^ as listed in Table 1.

**Table 1:** Clinical information and susceptibilities of LRSC isolates.

Isolate	Source	SCC*mec* type	MIC (mg L^−1^)															
			FOX	LNZ	CHL	SMZ	CIP	LEV	MXF	CLI	ERY	GEN	RIF	FOS	TET	VAN	TEC	DAP
LZD1	Blood	III	128	256	128	>8	16	8	4	>64	>256	>128	0.01	>1024	8	1	0.25	1
LZD2	Blood	III	128	256	128	>8	16	8	4	>64	>256	>128	0.01	>1024	8	1	0.25	1
LZD3	PICC catheter	III	128	256	128	>8	16	8	4	>64	>256	>128	0.01	>1024	8	1	0.25	1
LZD4	Blood	III	128	256	128	>8	16	8	4	>64	>256	>128	0.01	>1024	8	1	0.25	1
LZD5	Blood	III	128	256	128	>8	16	8	4	>64	>256	>128	0.01	>1024	8	1	0.25	1
LZD6	Blood	III	128	32	32	0.06	16	8	4	0.5	>256	>128	≤0.004	>1024	8	1	0.25	1
LZD7	PICC catheter	IV	128	32	64	0.125	16	8	2	>64	>256	64	≤0.004	>1024	2	1	0.25	1
LZD8	CVC catheter	III	128	256	256	0.06	16	8	4	>64	>256	>128	0.01	>1024	8	1	0.25	1
LZD10	CVC catheter	III	128	256	256	0.06	16	8	4	>64	>256	>128	0.01	>1024	8	1	0.5	1

Abbreviations: *FOX* cefoxitin, *LNZ* linezolid, *CHL* chloramphenicol, *SMZ* sulfamethoxazole/trimethoprim, *CIP* ciprofloxacin, *LEV* levofloxacin, *MXF* moxifloxacin, *CLI* clindamycin, *ERY* erythromycin, *GEN* gentamicin, *RIF* rifampin, *FOS* fosfomycin, *TET* tetracycline, *VAN* vancomycin, *TEC* teicoplanin, *DAP* daptomycin.

The whole genome sequence indicated that the *cfr*-carrying plasmid had clonal specificity. The hybrid assembly of LZD8 showed that this isolate contained three plasmids: pLZD8_1, pLZD8_2, and pLZD8_3. The *cfr* gene was carried by plasmid pLZD8_2. The result of the BLAST search on the NCBI GenBank showed that this *cfr*-carrying plasmid was highly homologous to plasmids pSR01(*S. aureus*), pXWZ_1(*S. capitis*), pH29-46(*S. aureus*), pLRSA47(*S. aureus*), and pSX01(*S. xylosus*). A comparison of the plasmids is shown in Supplementary Fig. 3. The three plasmids were detected among the examined genomes, and the results indicated that, unlike pLZD8_1, the plasmids pLZD8_2 and pLZD8_3 had strict clonal specificity, as they were detected only in clone L (Fig. 2).

The *cfr*-carrying plasmids from LZD1 and LZD8 were successfully conjugated to *S. aureus* 719 (ST5, *cfr* negative) but failed to conjugate to *S. aureus* ATCC29213-rifR. The transconjugants were named LZD1-719 and LZD8-719, both were identified as *S. aureus* and *cfr* positive. The transconjugant showed resistance to linezolid, and the resistance of chloramphenicol, gentamicin and clindamycin were also raised in these isolates, which can be explained by the gain of *cfr* gene (Supplementary Fig. 4). Revealed by linezolid E-test, the MIC of isolate 719 increased from 0.25 to 6 mg L^−1^ (LZD1-719) and 8 mg L^−1^ (LZD8-719).

Besides the presence of *cfr*, mutations in the 23S rRNA gene also contributed to linezolid resistance. The mutation of two linezolid resistance-related sites of 23S rRNA domain V, G2576T and C2104T, were detected in almost all the isolates with varied mutation percentages. All these L clone isolates harbored both mutations and the percentages of mutated copies ranged 66.7–100% and 50–100%, respectively. On the contrary, isolates from the other clones had relatively less percentages of mutated copies, with 0 to 29.4% mutated copies for C2104T and only one strain had G2576T mutation.

## Discussion

As an opportunistic pathogen, *S. capitis* causes severe bacteremia and device-related infections in adult ICUs and NICUs. Due to the lack of appropriate typing methods for *S. capitis*, little is known about its population structure. This impedes the advancement of research on this bacterium. Thus, the introduced typing method helps to determine the characteristics of multidrug-resistant *S. capitis* clones.

Generally, the core genome refers to genes shared among a certain collection of isolates of the same species. However, the cgMLST scheme contains a fixed set of genes conserved across the genome. Therefore, the simple detection of the core genome did not fit the cgMLST scheme requirements. Therefore, we used a validation set to modify the primary core genome. In addition, no standard workflow for establishing the cgMLST scheme has been generally accepted to date.

Previously, in the process of establishing a novel cgMLST scheme, owing to the algorithm of the core genome detecting software, the core genome was detected based on a reference genome^11,16^. Subsequently, the genes contained in the reference genome are filtered against those in numerous other genomes; if a gene is present in all other isolates, then it was included as a core gene. However, using this method, one may miss many genes that should have been included as core genes if they are absent in the reference genome. Conversely, the higher the number of core genes, the better the resolution of the scheme.

To detect as many core genes as possible, we applied the pangenome analysis software “Panaroo”, which is a graph-based pangenome clustering tool that accounts for many of the sources of error introduced during the annotation of prokaryotic genome assemblies. Due to the extra error correction and gene refinding steps, Panaroo detects more core genes than other software, such as PanX, Roary, and COGsoft^23^. Panaroo does not require a reference genome, avoiding the introduction of reference-bias in the downstream analysis.

Another important aspect of establishing a new cgMLST scheme is to include a validation step with an additional genome set. In the traditional method, only two sets are used. One is used to detect the core genome, and the other is used to test the scheme. This strategy overlooks the population structure and validation step. Here, we engaged three different sets: the primary set, validation set, and LRSC isolates. We chose assembly files from NCBI and EMBL-ENA as the primary set to take advantage of the wide span of time and geographical expanse. The structure of the primary set was more balanced than that of the validation set (Supplementary Fig. 5), considering that the NRCS-A clone comprised most of the validation set. Therefore, in our opinion, the primary set was suitable for detecting the primary core genome, and the validation set was suitable to modify the core genome. With the aid of sufficient genomes and Panaroo, the novel cgMLST scheme contained 1492 loci, which is considerably more than that of the cgMLST scheme created by Stenmark and colleagues^16^, even filtered with strict criteria. As a result, the novel cgMLST scheme provides more discriminatory power almost close to SNP-based phylogenetic analysis.

In the present study, using cgMLST analysis and phylogenetic analysis, we identified a unique clone, the L clone. Isolates recovered from various cities, across a span of many years, may not have a close genetic relationship; thus, the results indicate the spreading of the clone across hospitals in China. Similar to other pathogens, *Staphylococcus aureus*^24^, *Enterococcus faecium*^25^, and coxsackievirus^26^, LRSC may spread across cities, for example, Shanghai and Hangzhou.

The hybrid assembly of LZD8 showed that linezolid resistance was conferred by the *cfr*-carrying plasmid pLZD8_2. This plasmid was found to be clonal specific, only being carried by the L clone. The structure of this plasmid and the mobile element carrying *cfr* have been well described in another study^27^. This plasmid was first reported as pLRSA47 identified from six linezolid-resistant methicillin-resistant *S. aureus* (MRSA) isolates that belonged to ST5-II-t311 in the Second Affiliated Hospital of Zhejiang University in 2015^28^. However, early in 2013, an ST5 MRSA strain, named H29, isolated from the milk of hospitalized cattle in the United States^29^ contained the plasmid pH29-46, which was 99% identical to pLRSA47 and pLZD8_2. Another plasmid, pSX01 (KP890694), was detected in a *Staphylococcus xylosus* strain recovered from a pig in Henan, China in 2015. The other two plasmids, pSR01^30^ and pXWZ_1^31^ (KP890694), were detected in MRSA and *S. capitis* strains, respectively, as reported by our group. As shown in Supplementary Fig. 3, the comparison of these plasmids suggested that pLZD8_2 was distributed around the world from livestock farms to hospitals and caused linezolid resistance to spread among staphylococci. This was confirmed by the filter mating experiment from *S. capitis* to *S. aureus* in this study. However, the failure of filter mating experiment using ATCC29213-rifR strain indicated that this *cfr*-carrying plasmid could be host-specific.

Besides the presence of *cfr*, 23S rRNA mutations also contribute to linezolid resistance. Among the L clones, a few isolates did not carry the plasmid pLZD8_2 or *cfr* gene, but showed linezolid resistance (Supplementary Table 1). Using breseq, we mapped the genome reads to the 23S rRNA reference sequence and calculated the mutation proportion of each base. The mutation detection result indicated that the L clone is characterized by multiple copies of C2576G and C2104T mutations, in accordance with the findings of a previous study^17^. Based on this evidence, we inferred that the L clone might have been previously exposed to anti-ribosomal drugs such as linezolid or florfenicol, which are often used in the ICU. However, we do not have sufficient evidence to trace its origin.

The L clone contained more drug-resistant genes than other clones; thus, focus on this clone is required. Multiple antibiotic resistance of bacteria has led to increased morbidity and mortality, as well as increased adverse outcomes^32^. Resistance to antibiotics causes the L clone to successfully spread and persist in different ICUs. In addition, the extra resistance genes and plasmids result in a higher fitness cost^33^ than that of the NRCS-A clone, which is probably the reason that the L clone is not predominant.

According to previous epidemiological investigations^34^, the linezolid resistance rates of staphylococci were low, indicating that sporadic linezolid resistant staphylococci infection might not be a real threat in clinical settings. However, our results showed that the spread of the L clone was probably underestimated. With the help of the *S. capitis* cgMLST scheme, we are able to determine the actual role of the L clone in spreading multiple drug resistance. International surveillance projects are needed to detect the intercontinental spread of LRSC.

In conclusion, we have established a reliable cgMLST scheme for *S. capitis*, with a high resolution close to that of an SNP-based phylogenetic analysis. Using this scheme, we detected a widespread multidrug-resistant clone, and labeled it the L clone. Further epidemiological investigation is needed, and it is worth investigating the L clone to stop the further spread of drug resistance.

## Methods

### Ethics issues

This study was approved by the ethics committee of Sir Run Run Shaw Hospital (No. 20210319-33). Informed consent was waived, as the study used only anonymized clinical data unlinked to patient identifiers, and data produced in this study was not used for the treatment or management of patients.

### Establishment and modification of the *S. capitis* cgMLST scheme

The workflow of scheme development is presented in Supplementary Fig. 1. First, we collected all available genome assemblies of *S. capitis* in the NCBI GenBank database and the European Nucleotide Archive of European Molecular Biology Laboratory (EMBL-ENA) as of June 11, 2021 using SRA Toolkit (https://github.com/ncbi/sra-tools). A total of 142 genome assemblies, submitted from February 3, 2009 to May 19, 2021, were collected. After performing average nucleotide identity analysis (ANI) with pyANI^35^ (version 0.2.10), assembly quality control with panaroo-qc^23^, 136 genome assemblies were obtained, and they are listed in Supplementary Data 1; these assemblies served as the primary genome set.

After annotated using Prokka^36^, core genome analysis was performed using this primary genome set to obtain the primary cgMLST scheme. Core genes were detected with Panaroo^23^ (version 1.2.8) and filtered based on the following criteria: (1) discarding all genes that did not contain a start codon at the beginning of the gene; (2) discarding all genes that contained more than one stop codon or those that did not have a stop codon at the end of the gene; (3) discarding the genes that were shorter than 50 bp; (4) discarding the potential paralogs by comparing each locus against all alleles using the Basic Local Alignment Search Tool (BLAST)^37^ (version 2.9.0+), with an identity of 0.9; (5) discarding the shorter gene if two genes were affected by an overlap of >3 bp on the reference chromosome CR01 (accession number LN866849); (6) collecting plasmids of *S. capitis* with Ridom SeqSphere^+^ software version 7.2.3^38^ (Ridom GmbH, Muenster, Germany) (search date: June 18, 2021) and discarding genes homologous to those genes contained within plasmids; and (7) filtering genes that were homologous with the transposon_db in the TransposonPSI database^39^ (version 1.0.0).

Next, we modified the remaining core genes using a validation genome set. The validation genome set was collected from an international study of *S. capitis*^7^, consisting of 250 isolates from 22 countries worldwide, collected between 1994 and 2015. The raw reads (Fastq files) of the collection were downloaded from the NCBI Sequence Read Archive (SRA), with BioProject accession number PRJNA493527. We reassembled the genomes using Shovill (version 2.0.3, T. Seeman, unpublished, https://github.com/tseemann/shovill), and typed with the primary cgMLST scheme to acquire allelic profiles. The genes with error rates greater than 5% were removed from the primary cgMLST target genes^40^, resulting in the final version of the cgMLST scheme.

### Evaluation of the cgMLST scheme

To validate the ability of the cgMLST scheme to cluster related isolates, we imported the genomes of the validation set to create a minimum spanning tree with the Ridom default setting, disregarding the missing data in the pairwise comparisons. Isolates with less than 24 allelic differences were considered to be the related isolated groups^41^.

Phylogenetic analysis of the validation genome set was performed to assess the population structure. Based on the core genome aligned sequences, IQ-TREE^42^ (version 2.0.3) was used to construct a single-nucleotide polymorphism (SNP)-based phylogenetic tree. The phylogenetic tree was visualized and labeled using the iTOL^43^ Web service.

### Application of cgMLST to LRSC typing

To evaluate the applicability of the *S. capitis* cgMLST scheme for outbreak analysis, we reanalyzed the published genomic data of *S. capitis* isolates from an outbreak in Shanghai^17^ and another independent outbreak in Sir Run Run Shaw Hospital in Hangzhou as well as two sporadic cases, one in Harbin and one in Hangzhou. All genomes included are listed in Supplementary Table 1.

Nine LRSC isolates recovered from Sir Run Run Shaw Hospital from May 2016 to April 2017 were included in this study. Antibiotic susceptibility testing (AST) of common drugs were performed using agar or broth dilution methods according the recommendations of Clinical and Laboratory Standards Institute (CLSI)^44^. The genomes of those isolates were sequenced using a HiSeq X Ten platform (Illumina, San Diego, CA) with 2 × 150 bp paired-end reads. The isolate LZD8 was randomly selected and sequenced using nanopore sequencing. The complete genome of LZD8 was constructed using hybrid assembly of short and long read sequences using Unicycler^45^ (version 0.4.8). The quality of the fastq files was examined using FastQC^46^ (version 0.11.9) and MultiQC^47^ (version 1.10.1). Assembly and annotation were performed using Shovill and Prokka^36^ (version 1.14.6). The clinical information of these nine isolates was collected. To evaluate the antibiotic pressure, the consumption of linezolid in our hospital was assessed using AUD (defined daily dose per 100 patient-days).

### Genomic typing and resistance analysis

SCC*mec* typing was performed on all *S. capitis* genomes using SCCmecFinder on the CGE website (https://cge.cbs.dtu.dk/services/SCCmecFinder/). Resistance genes were detected using ABRicate (version 1.0.0, https://github.com/tseemann/abricate). The existence of plasmids was detected in assembly files using BLAST. The mutation of 23S rRNA domain V in the isolates having an acquirable fastq sequence file was detected using breseq^48^ (version 2.0.3). All detected features were labeled using a heatmap or with color stripes around the phylogenetic tree.

### Filter mating experiments

Filter mating experiments were performed to investigate whether the *cfr* carrying plasmid is conjugative. Using a clinical isolate *S. aureus* 719 (ST5, *cfr* negative) and ATCC29213-rifR as recipients and *S. capitis* LZD1 and LZD8 as donors with selection on nutrient agar plates containing 4 mg L^−1^ linezolid and 12.5 mg L^−1^ tetracycline or plates containing 4 mg L^−1^ linezolid and 50 mg L^−1^ rifampin according to the reference with adjustment in antibiotic concentrations^49,50^. The transconjugants were identified using MALDI-TOF and PCR of *cfr* gene with primers (*cfr*-fw: TGAAGTATAAAGCAGGTTGGGAGTCA; *cfr*-rv: ACCATATAATTGACCACAAGCAGC)^51^. Thereafter, antibiotic susceptibility test (K-B test and E-test) was performed to assess the change in drug resistance.

### Reporting summary

Further information on research design is available in the Nature Research Reporting Summary linked to this article.

## Supplementary information

Supplementary Information

Description to Additional Supplementary Information

Supplementary Data 1

Reporting Summary

## Data Availability

The genomes that included in the primary genome set were collected from public databases and those accession numbers were listed in Supplementary Data 1. The validation set was retrieved with BioProject accession number PRJNA493527^7^. As for the linezolid-resistant strains isolated in Sir Run Run Shaw Hospital, the assembly files can be downloaded using the BioProject number PRJNA748212, and the complete genome of LZD8 can be downloaded with the GenBank accession number SAMN23101375. The genome of isolate XWZ can be downloaded with the accession number SAMN23101376. All data obtained or analyzed in this study underlying the figures in this manuscript are available in Supplementary Data 1 or in the Source Data file. Source data are provided with this paper Source data are provided with this paper.

## Source data

Source Data

## Code availability

Computer code is available from GitHub under https://github.com/gooday92/cgMLST_s_capitis.
