# Supplementary material for: Development of a novel core genome MLST scheme for tracing multidrug resistant Staphylococcus capitis
Source: Nat Commun. 2022 Jul 22;13:4254. doi: 10.1038/s41467-022-31908-x (PMC9307846; doi:10.1038/s41467-022-31908-x)
Supplement: Supplementary file 1 — Supplementary Information [file 41467_2022_31908_MOESM1_ESM.pdf]

Development of a novel core genome MLST scheme for tracing multidrug resistant *Staphylococcus capitis*

Zhengan Wang[1,2,3,†], Chao Gu[1,2,3,4,†], Lu Sun[1,2,3,†], Feng Zhao[5,6], Ying Fu[2,3,5,6], Lingfang Di[2,3,7], Junxiong Zhang[2,3,8], Hemu Zhuang[1,2,3], Shengnan Jiang[1,2,3], Haiping Wang[1,2,3], Feiteng Zhu[1,2,3], Yiyi Chen[1,2,3], Mengzhen Chen[1,2,3], Xia Ling[1,2,3,9], Yan Chen[1,2,3,*], Yunsong Yu[1,2,3,*]

Correspondence to: Yunsong Yu, yvys119@zju.edu.cn; Yan Chen, chenyan@zju.edu.cn.

This PDF file includes:

- Supplementary Figures 1 to 5.
- Supplementary Table 1

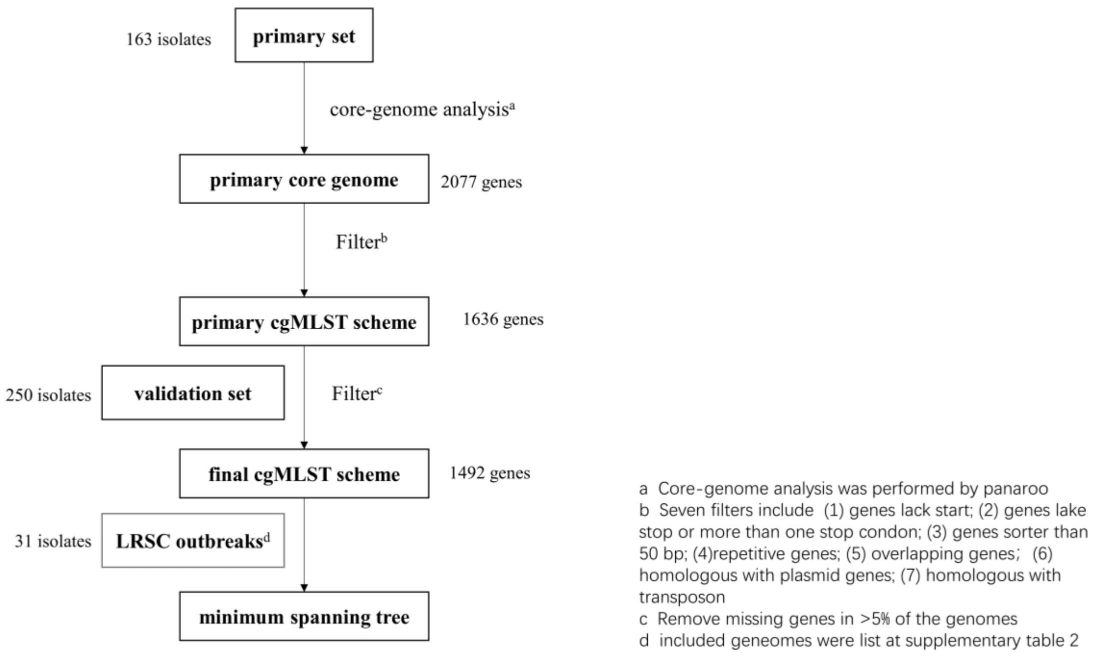

Supplementary Fig. 1: The workflow of the establishment of *Staphylococcus capitis* core genome multilocus sequence typing (cgMLST) scheme.

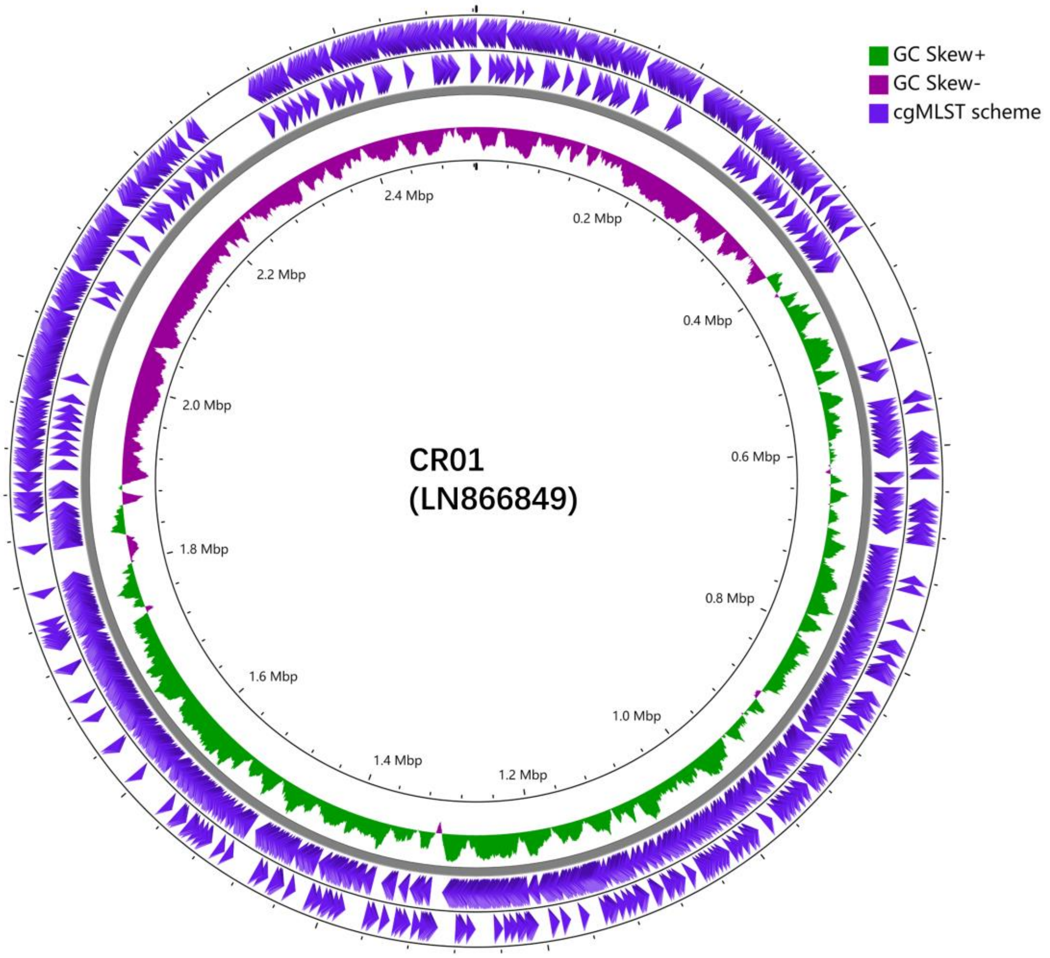

Supplementary Fig. 2: The distribution of cgMLST scheme on CR01 chromosome.

The almost final cgMLST scheme, which is 1,491 genes, are marked on the CR01 chromosome using CGview (http://cgview.ca/).

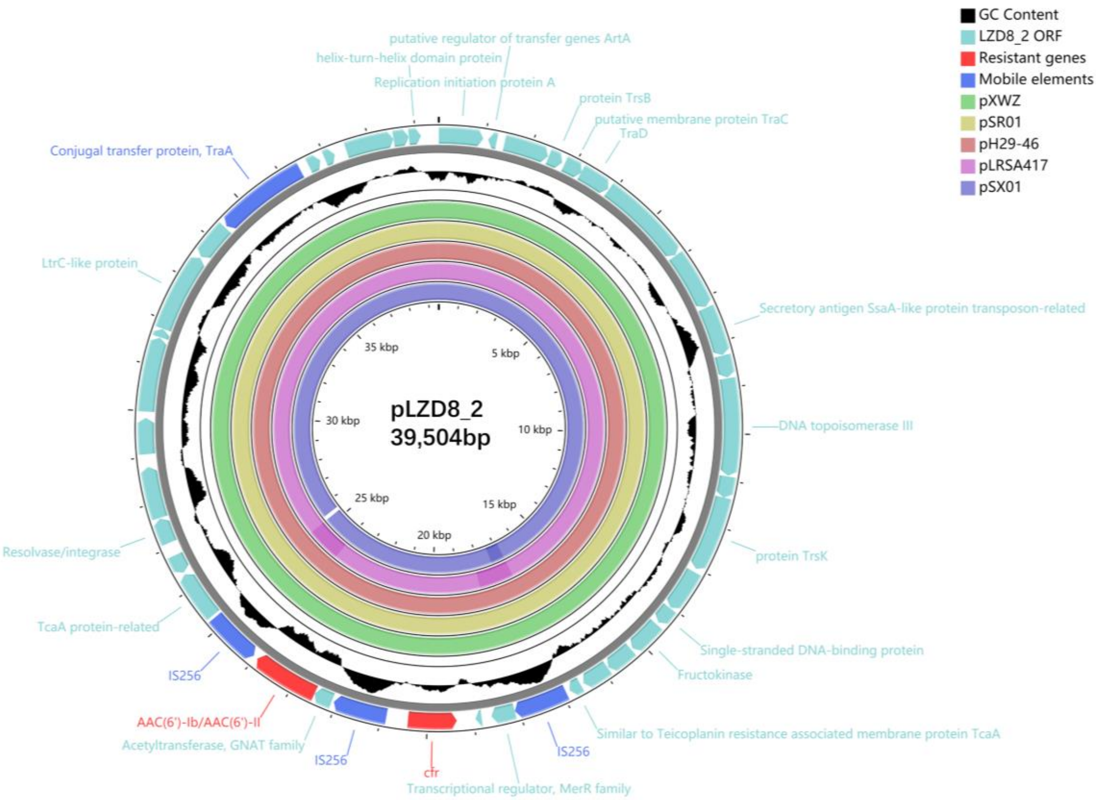

Supplementary Fig. 3: Comparison of *cfr*-carrying plasmids.

With pLZD8_2 as the map, 5 other *cfr*-carrying plasmids were compared, including pXWZ, pSR01, pH29-46, pLRSA417, and pSX01, using CGview (http://cgview.ca/). Mobile elements are marked in purple color; resistant genes are marked in red color and the rest genes were marked with light blue.

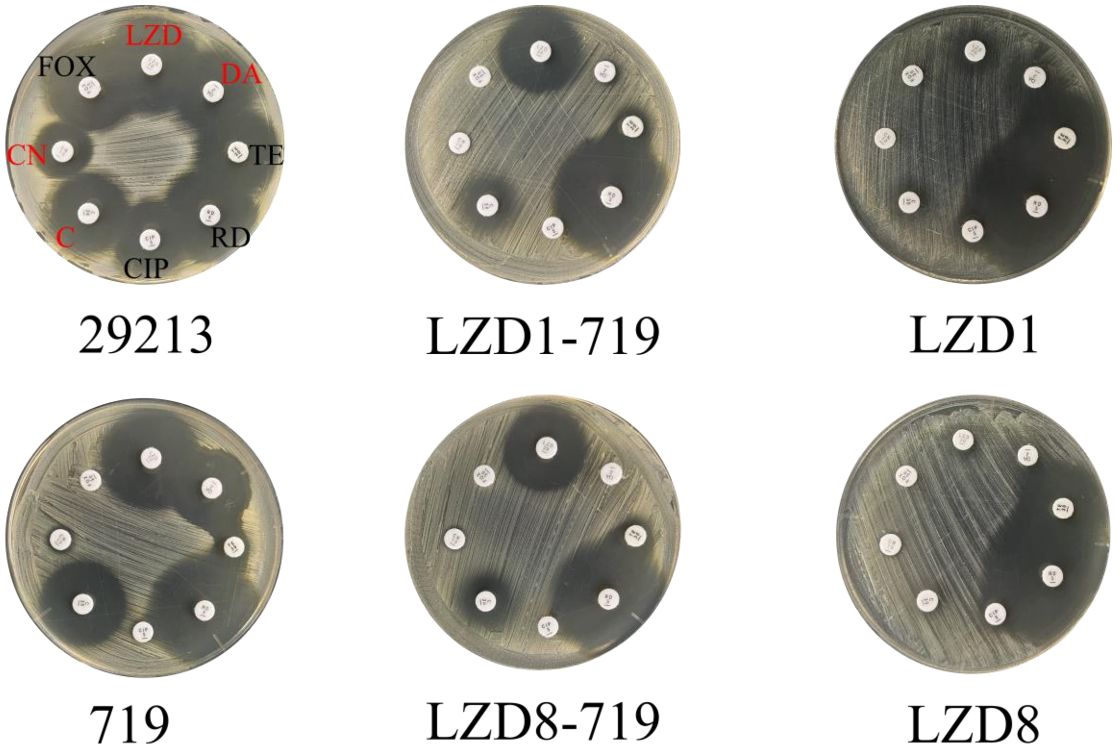

Supplementary Fig. 4: Antibiotic susceptibility test (K-B) result of filter mating experiments.

Eight drugs were tested which include cefoxitin (FOX), linezolid (LZD), clindamycin (DA), tetracycline (TE), rifampicin (RD), ciprofloxacin (CIP), chloramphenicol (C) and gentamycin (CN). *S. aureus* 29213 was control, *S. aureus* 719 was recipient, *S. capitis* LZD1 and LZD8 were donors, LZD1-719 and LZD9-719 were the transconjugants. The position of drugs on the six plates were the same. The red drug names indicated changed resistance between recipient and transconjugant.

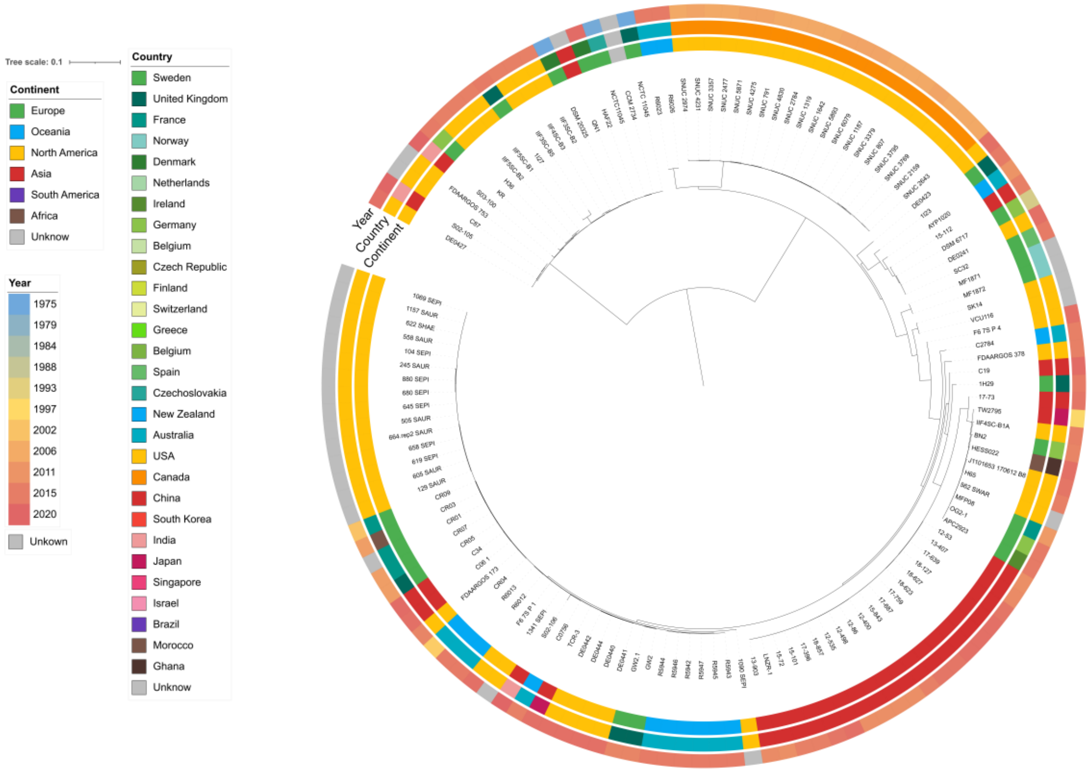

Supplementary Fig. 5: The phylogenetic tree of the primary set.

The phylogenetic tree is constructed with the same way as the phylogenetic tree of the validation set and LRSC which has been described. The color strips around the tree indicate the isolated continent, isolated country and isolated year.

Supplementary Table 1: Information of linezolid-resistant *Staphylococcus capitis* strains include in this study.

Isolate	*cfr* gene	Isolate city	Isolate year
LZD1	+	Hangzhou	2016
LZD2	+	Hangzhou	2016
LZD3	+	Hangzhou	2016
LZD4	+	Hangzhou	2016
LZD5	+	Hangzhou	2017
LZD6	-	Hangzhou	2016
LZD7	-	Hangzhou	2016
LZD8	+	Hangzhou	2017
LZD10	+	Hangzhou	2017
XWZ	+	Hangzhou	2008
LNZR-1	+	Harbin	2012
12-53	+	Shanghai	2012
12-86	+	Shanghai	2012
12-400	+	Shanghai	2012
12-498	+	Shanghai	2012
12-535	-	Shanghai	2012
13-407	+	Shanghai	2013
13-903	-	Shanghai	2013
15-72	-	Shanghai	2015
15-101	-	Shanghai	2015
15-843	-	Shanghai	2015
17-84	-	Shanghai	2017
17-124	-	Shanghai	2017
17-396	+	Shanghai	2017
17-639	-	Shanghai	2017
17-687	+	Shanghai	2017
17-759	+	Shanghai	2017
18-127	+	Shanghai	2018
18-623	+	Shanghai	2018
18-627	+	Shanghai	2018
18-857	+	Shanghai	2018
