# Supplementary material for: Development of a novel core genome MLST scheme for tracing multidrug resistant Staphylococcus capitis
Source: Nat Commun. 2022 Jul 22;13:4254. doi: 10.1038/s41467-022-31908-x (PMC9307846; doi:10.1038/s41467-022-31908-x)
Supplement: Supplementary file 2 — Description to Additional Supplementary Information [file 41467_2022_31908_MOESM2_ESM.pdf]

Description of Additional Supplementary Files

Supplementary Data 1. The metadata of the primary genome set. The assembled genomes of Staphylococcus capitis that formed the primary genome set which were collected from public databases after quality control.
