# Supplementary material for: Development of a novel core genome MLST scheme for tracing multidrug resistant Staphylococcus capitis
Source: Nat Commun. 2022 Jul 22;13:4254. doi: 10.1038/s41467-022-31908-x (PMC9307846; doi:10.1038/s41467-022-31908-x)
Supplement: Supplementary file 4 — Reporting Summary [file 41467_2022_31908_MOESM4_ESM.pdf]

nature portfolio

Corresponding author(s): Yunsong Yu, Yan Chen

Last updated by author(s): May 2, 2022

Reporting Summary

Nature Portfolio wishes to improve the reproducibility of the work that we publish. This form provides structure for consistency and transparency in reporting. For further information on Nature Portfolio policies, see our Editorial Policies and the Editorial Policy Checklist.

Statistics

For all statistical analyses, confirm that the following items are present in the figure legend, table legend, main text, or Methods section.

n/a	Confirmed	
x		The exact sample size (*n*) for each experimental group/condition, given as a discrete number and unit of measurement
x		A statement on whether measurements were taken from distinct samples or whether the same sample was measured repeatedly
x		The statistical test(s) used AND whether they are one- or two-sided<br>*Only common tests should be described solely by name; describe more complex techniques in the Methods section.*
x		A description of all covariates tested
x		A description of any assumptions or corrections, such as tests of normality and adjustment for multiple comparisons
	x	A full description of the statistical parameters including central tendency (e.g. means) or other basic estimates (e.g. regression coefficient) AND variation (e.g. standard deviation) or associated estimates of uncertainty (e.g. confidence intervals)
x		For null hypothesis testing, the test statistic (e.g. *F*, *t*, *r*) with confidence intervals, effect sizes, degrees of freedom and *P* value noted<br>*Give P values as exact values whenever suitable.*
x		For Bayesian analysis, information on the choice of priors and Markov chain Monte Carlo settings
x		For hierarchical and complex designs, identification of the appropriate level for tests and full reporting of outcomes
x		Estimates of effect sizes (e.g. Cohen's *d*, Pearson's *r*), indicating how they were calculated

Our web collection on statistics for biologists contains articles on many of the points above.

Software and code

Policy information about availability of computer code

Data collection	Using SRA Toolkit (2.8.0) to download fastq genomes of Staphylococcus capitis from NCBI.
Data analysis	Computer code is available from GitHub under https://github.com/gooday92/cgMLST_s_capitis. Other used softwares include: FastQC (0.11.9), MultiQC (1.10.1), Shovill (0.9.0), Prokka (1.14.6), pyANI (0.2.10), Panaroo (1.2.8), BLAST (2.9.0+), IQ-TREE (2.0.3), TransposonPSI (1.0.0), ABRIcate (1.0.0), breseq (0.34.1), Unicycler (0.4.8), Ridom SeqSphere+ software(7.2.3), SCCmecFinder on the CGE website, CGview and iTOL web service.

For manuscripts utilizing custom algorithms or software that are central to the research but not yet described in published literature, software must be made available to editors and reviewers. We strongly encourage code deposition in a community repository (e.g. GitHub). See the Nature Portfolio guidelines for submitting code & software for further information.

Data

Policy information about availability of data

All manuscripts must include a data availability statement. This statement should provide the following information, where applicable:

- Accession codes, unique identifiers, or web links for publicly available datasets
- A description of any restrictions on data availability
- For clinical datasets or third party data, please ensure that the statement adheres to our policy

The genomes that were included in the primary genome set were collected from public databases and are listed in Supplementary Table 1. The validation set was available with BioProject accession number PRJNA493527. As for the linezolid-resistant strains isolated in Sir Run Run Shaw Hospital, the assembly files can be downloaded using the BioProject accession number PRJNA748212, and the complete genome of LZD8 can be downloaded with the GenBank accession number SAMN23101375. The genome of isolate XWZ can be downloaded with the accession number SAMN23101376.

Field-specific reporting

Please select the one below that is the best fit for your research. If you are not sure, read the appropriate sections before making your selection.

- [x] Life sciences
- [ ] Behavioural & social sciences
- [ ] Ecological, evolutionary & environmental sciences

For a reference copy of the document with all sections, see nature.com/documents/nr-reporting-summary-flat.pdf

Life sciences study design

All studies must disclose on these points even when the disclosure is negative.

Sample size	All linezolid resistant Staphylococcus capitis isolates were collected which was 9 isolates.
Data exclusions	Genomes failed in quality control were excluded, which was performed using pyANI and panaroo-qc.
Replication	We chose two isolates of LRSC as donors to perform the filter mating experiments three times and each time we got the same result. Only on the situation of Staphylococcus capitis as donor, Staphylococcus aureus 719 as recipients and selecting on nutrient agar plates containing 4 mgL−1 linezolid and 12.5 mgL−1 tetracycline, we got positive result.
Randomization	We did not divide experimental groups or control groups.
Blinding	In this study, blinding was unnecessary since result were verified with several objective methods.

Reporting for specific materials, systems and methods

We require information from authors about some types of materials, experimental systems and methods used in many studies. Here, indicate whether each material, system or method listed is relevant to your study. If you are not sure if a list item applies to your research, read the appropriate section before selecting a response.

Materials & experimental systems

n/a	Involved in the study	
x		Antibodies
x		Eukaryotic cell lines
x		Palaeontology and archaeology
x		Animals and other organisms
x		Human research participants
x		Clinical data
x		Dual use research of concern

Methods

n/a	Involved in the study	
x		ChIP-seq
x		Flow cytometry
x		MRI-based neuroimaging
